# K3326X and Other C-Terminal BRCA2 Variants Implicated in Hereditary Cancer Syndromes: A Review

**DOI:** 10.3390/cancers13030447

**Published:** 2021-01-25

**Authors:** Scott Baughan, Michael A. Tainsky

**Affiliations:** 1 Department of Oncology, Wayne State University School of Medicine, Detroit, MI 48201, USA; sbaughan@med.wayne.edu; 2 Center for Molecular Medicine and Genetics, Wayne State University School of Medicine, Detroit, MI 48201, USA

**Keywords:** hereditary breast and ovarian cancer syndrome, HBOC, hereditary breast ovarian and pancreatic cancer syndrome, HBOPC, BRCA2, K33326X

## Abstract

**Simple Summary:**

The cancer associated protein BRCA2 is the subject of intense continual study. Because of this, new insights into the relation of specific variants of this gene and cancer are regularly generated. These discoveries shed light on cancer risk and management for patients carrying these mutations. Additionally, new techniques for variant discovery and investigation are developed and tested, further enhancing scientific and clinical understanding of this key protein. In this review we will investigate the recent literature associated with variants in the C-terminus of BRCA2 and their effect on health and cancer predisposition.

**Abstract:**

Whole genome analysis and the search for mutations in germline and tumor DNAs is becoming a major tool in the evaluation of risk as well as the management of hereditary cancer syndromes. Because of the identification of cancer predisposition gene panels, thousands of such variants have been catalogued yet many remain unclassified, presenting a clinical challenge for the management of hereditary cancer syndromes. Although algorithms exist to estimate the likelihood of a variant being deleterious, these tools are rarely used for clinical decision-making. Here, we review the progress in classifying K3326X, a rare truncating variant on the C-terminus of BRCA2 and review recent literature on other novel single nucleotide polymorphisms, SNPs, on the C-terminus of the protein, defined in this review as the portion after the final BRC repeat (amino acids 2058–3418).

## 1. Introduction

The central role BRCA2 plays in human genome stability makes it a key player in hereditary cancers. Despite decades of work, many variants of unknown clinical significance exist throughout the protein, providing a challenge for clinicians counseling patients with these variants. Indeed, most patients who undergo testing for a familial cancer pattern will have one or more variants of unknown significance (VUS), and this phenomenon results in indefinite guidance for patient risk management.

Due to its role in cancer initiation and progression, BRCA2 loss of function variants play a key role in determining patient prognosis and treatment patterns. Tumors deficient in BRCA proteins show enhanced susceptibility to PARP inhibitor therapy combined with platinum salts [1,2]. It is therefore important to study and understand the hereditary mutations affecting the BRCA2 protein throughout the human population in order to increase the ability of clinicians to predict and treat cancers related to hypomorphic or nonfunctional BRCA2 alleles. Understanding these variants will play a key role in delivering precision medicine to patients carrying such mutations.

## 2. The Homology Directed Repair Pathway

BRCA2 is a key mediator of DNA double-strand break repair in animals. Two possible pathways exist through which double-strand breaks can be repaired: nonhomologous end joining (NHEJ) and homologous recombination (HR or HDR). BRCA2 is a key participant in the HDR repair pathway (reviewed in [3]).

HDR begins with the recognition of double-strand breaks by phosphorylation and activation of the ATM kinase, in part through the action of the MRN complex [4,5]. ATM then proceeds to phosphorylate dozens of targets, including histone H2AX and NBS1 [4,6,7,8,9,10]. NBS1 interacts with MRE11 and Rad50, forming the MRN complex that brings the two DNA ends of the double strand break into proximity and holds them together, preventing degradation [8,11]. The MRN complex, along with CtIP, trims the 5′ broken ends, after which RPA is recruited to the site of the double strand break, stabilizing the resulting single stranded DNA [12,13,14]. Phosphorylation of BRCA2 by Chk1, Chk2, and CDK1 enables the binding of BRCA2 to Rad51 and exposure of nuclear localization signals on BRCA2 and binding of PALB2 to BRCA2 enables the attachment of BRCA2 to DNA (discussed in depth below). This step brings the BRCA2-Rad51 complex into the nucleus, after which the action of BRCA2′s BRC repeats and Rad51-nucleofilament binding domain facilitate the displacement of RPA, and, by different affinity for Rad51 and ssDNA and high affinity for the Rad51-ssDNA complex, the formation of the Rad51-ssDNA complex [15,16]. This allows Rad51 to complete the role of initiating the search for homology and single-strand invasion [17,18]. PCNA holds the strands in place while the missing DNA is then replicated by cellular DNA replication machinery, after which the strands are decoupled and the backbones re-ligated [19,20,21].

## 3. BRCA2 Structure and Biochemical Functions

BRCA2 plays a complex and multifaceted role in DNA repair, with numerous interactions and binding domains influencing diverse cellular outcomes. Loss of BRCA2 promotes genomic instability, with large deletions and amplifications [22,23]. BRCA2-null mice have an embryonic lethal phenotype [24], and biallelic germline mutations in BRCA2 are rare, but may result in a Fanconi anemia [25]. BRCA2 is critical for the initiation of DNA repair and the coordination and regulation of the diverse functions of Rad51 in the DNA damage response though its multiple RAD51 interacting domains (BRC repeats 1–8), which function to recruit Rad51 to the site of double-strand breaks [22,23,25,26].

The complete structure of the BRCA2 gene was first published in 1996, spanning 27 exons on 70 kb of genomic DNA, resulting in a 3418 amino acid protein with a molecular weight of 384 kDa [27]. BRCA2 has an N-terminal transactivation domain spanning residues 18–105. PALB2 binds the N-terminus of BRCA2 and assists with DNA binding and promotion of Rad51 D-loop formation [26]. Failure of D-loop formation leads to the hypersensitivity to certain DNA damage-causing drugs seen with BRCA2 deficiency [25].

The central section of the protein is composed of eight 34 amino acid repeats termed “BRC repeats” interspersed from amino acid 1002 to 2085. BRCA2 has two distinct groups of BRC repeat motifs: BRC repeats 1–4 and BRC repeats 5–8. BRC repeats 1–4 inhibit Rad51-ssDNA ATPase activity, bind free Rad51, and prevent Rad51 binding of dsDNA. Because Rad51 has less affinity for ssDNA than RPA, BRCA2 is essential in enabling Rad51 to displace RPA, an action that involves the binding of free Rad51 by the first four BRC repeats [23,26,28]. BRCA2 stabilizes the nucleofilament by maintaining the active form of Rad51, leading to the exchange of Rad51 and RPA [28]. Importantly, the C-terminus of BRCA2 directly interacts with DNA and RAD51, preventing disassembly of the nucleofilament by earlier BRC repeats [29]. Together with the DNA binding domains, BRC repeats 5–8 facilitate the binding of Rad51 to ssDNA and have increased affinity for the nucleoprotein filament rather than the free protein, promoting stabilization of the already formed complex, thus facilitating the Rad51-mediated strand exchange and invasion necessary for homology-dependent repair [28]. Successful loading of Rad51 also requires the actions of the Rad51 paralogues, though their exact function is not currently clear [30]. Altogether, BRCA2 acts to first bind free Rad51, enabling complex formation, then to bind and stabilize the complex at the site of DNA damage, allowing for Rad51′s exchange with RPA and Rad51-mediated interstrand invasion and homology search, all while preventing unnecessary action by Rad51 which could have mutagenic consequences [25,26,28,29,30]. BRCA2 also facilitates the loading of Rad51 onto telomeres, protecting chromosome ends and is necessary for the repair of interstrand crosslinks [25].

Multiple studies have shown that the C-terminus of BRCA2 is essential for proper nuclear localization of the protein, containing two NLS present in the last 156 amino acids (AA 3263 to 3269 and A 3381 to 3385) [31,32]. Chk1 and Chk2 are known to phosphorylate T3387 of BRCA2, likely modifying the NLS at the C-terminus and controlling localization of BRCA2 to the nucleus. T3387 is also involved in the retention and release of Rad51, with failure of phosphorylation at this site preventing accumulation of Rad51 at nuclear foci. Phosphorylation of S3291 by CDK1 additionally regulates Rad51 binding to the BRCA2 C-terminus [33].

BRCA2 also directly interacts with p53 through a binding site located on the C-terminal OB2 and OB3 domains (amino acids 2669–3051) [34]. The final Rad51 binding site, spanning 3265–3330, is phosphorylated by CDK1, indicating that interaction with Rad51 and BRCA2 is regulated by CDK1. This site has a preference for Rad51 oligomers, assisting in the formation of Rad51, ssDNA complex formation [3]. The C terminus DNA-binding domains OB1–OB3, in combination with the nearby Rad51 binding site, are thought to protect the Rad51 nucleofilament from disassembly by the earlier BRC repeats, stabilizing the nucleofilament [2,26,29]. This site is also essential for protecting the Rad51 nucleofilament from MRE11-mediated degradation, and protecting stalled replication forks from collapse [23,25,26,35]. This role is independent from BRCA2′s primary function in HDR and requires S3291 [28]. Finally, the C-terminus of BRCA2 contains a binding site for DMC1 binding, essential for the interaction of the two proteins [36,37], and a nuclear export sequence at 2682–2698, which is masked by DSS1 for proper function [38].

**Figure 1:**
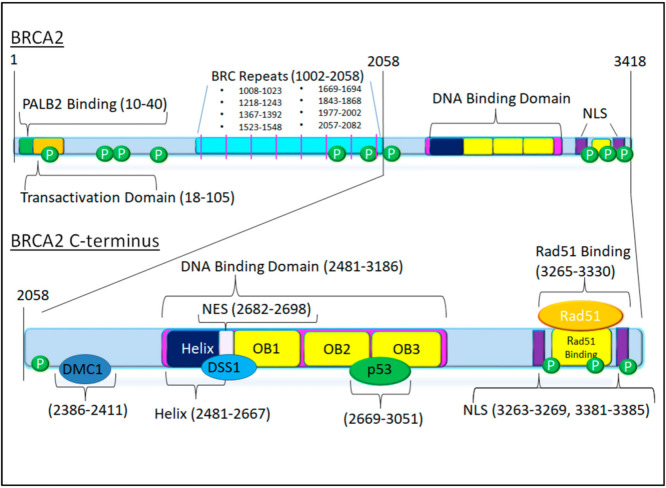
The BRCA2 Protein. The C-terminus of the protein (shown expanded in the bottom of the figure) from amino acid 2058, immediately after the BRC repeats, to the end of the protein, amino acid 3418. The C-terminus contains several features of note, including a DMC1 binding site, a DNA-binding domain in which there is a nuclear export sequence masked by DSS1 binding as well as a p53 binding site, and a Rad51 binding site. Two nuclear localization signals flank the Rad51 binding site. Phosphorylation sites are present at S2095, S3291, S3319, and T3387.

A map of the domains present on the C-terminus of BRCA2 is shown in Figure 1. These mechanisms point to the essential role of the C-terminus in the numerous functions of BRCA2.

**Figure 2:**
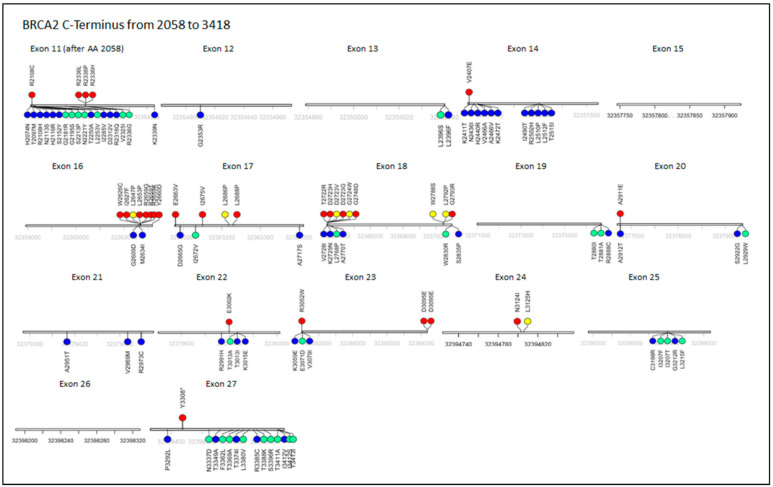
BRCA2 C-terminal variants: ClinVar data for missense mutations in the C-Terminus of BRCA2. Top: Red = Pathogenic, Yellow = Likely Pathogenic. Bottom: Green = Likely Benign, Blue: Benign. The most distal known pathogenic truncation is shown on Exon 27. Data on the pathogenicity of missense mutations can show hot and cold spots in the protein, guiding further inquiry and prioritizing some mutations for study. Over 4600 missense variants of unknown significance (VUS) exist in BRCA2 at the time of writing, with 1833 of those on the C-terminus (after the BRC repeats at Amino Acid 2058).

The vast majority of missense variations in BRCA2 are of unknown significance, and too few are classified to identify true hotspots [39]. Figure 2 shows a graphical representation of known pathogenic and benign missense mutations on the C-terminus of BRCA2, and well as the distal most truncation known to be pathogenic.

## 4. The K3326X Mutation

One of the most prolifically studied and controversial C-terminal mutations of BRCA2 is K3326X, in which a lysine is mutated, resulting in early truncation of the protein with final 93 amino acids being lost. The lost domains include Thr3387, essential for the release of Rad51, the most C-terminal nuclear localization signal, and a portion of the distal Rad51-ssDNA as well as the DMC1 binding domains [2,22,25,29,34,36]. Recent evidence indicates that BRCA2 may play a key role in the repair and recovery of the cell from stalled replication forks and that exon 27, in which the K3326X mutation occurs, is essential in this function [23,30,35,40,41].

### 4.1. K3326X in Gynecologic Cancers

K3326X is well-investigated with regard to gynecologic cancer risk. A landmark study using over 70,000 cancer cases and 80,000 controls showed increased risk of breast (OR = 1.28), invasive ovarian (OR = 1.26), serous ovarian (OR = 1.46), and ER-negative breast cancer (OR = 1.5) in K3326X mutation carriers. Additionally, for individuals with a second mutation in BRCA1, there was an increased risk of ovarian cancer, showcasing the potential for additional pleiotropic events with this variant [42]. As Arbustini and colleagues note in a follow-up to this article, these new data change the knowledge paradigm regarding K3326X. They recommend expanded counseling for women with K3326X and hypothesize that these patients may be good candidates for PARP inhibitor therapy due to BRCA2 dysfunction [43]. The case study of an Italian family further implicated the K3326X variant in the development of early onset cancer and recommends that the K3326X mutation be evaluated with other pathogenic mutations [44]. Data from mouse models further supports this idea: mice lacking the final exon of BRCA2 are viable but show increased tumor incidence compared to normal littermates [45]. Finally, a 2017 cohort study found higher than expected prevalence of the K3326X allele in individuals with a history of familial cancer and a personal history of ovarian cancer (OR = 4.95, *p* = 0.01) [46]. Citing additional data from the Ovarian Cancer Association Consortium, the authors hypothesize that BRCA2 K3326X is likely a low risk allele for ovarian cancer with an OR of 1.22–9.3.

### 4.2. K3326X in Pancreatic Cancer

In addition to gynecological cancers, BRCA2 K3326X was investigated in the context of familial pancreatic cancer though the study of 250 patients with sporadic pancreatic cancer, 114 patients with familial pancreatic adenocarcinoma, 115 spouses of patients with pancreatic cancer as an additional environmental control, and a second control group of 125 patients with no cancer history undergoing cholecystectomy for other reasons. In individuals with familial pancreatic cancer, K3326X was present at a much higher frequency of 5.6%, compared to 1.2% in controls (OR = 4.84, 95% CI 1.27–18.55, *p* < 0.01). There was no association between the mutation and sporadic pancreatic cancer (OR: 2.37, 95% CI 0.61–9.27) [47]. Similarly, K3326X was investigated in a case control study of 5626 control subjects and 2935 sporadic cases of pancreatic ductal adenocarcinoma. The authors found an association with the K3326X variant with an OR = 1.78 (95% CI = 1.26–2.52, *p* = 1.19 × 10^−3^). The odds ratio remained significant when controlling for family history. K3326X was not associated with pancreatic ductal adenocarcinoma with onset before 50 years of age (OR = 1.87, 95% CI = 0.93–3.74, *p* = 0.08) [48].

### 4.3. K3326X in Environmental Cancers

BRCA2 K3326X has been intensively studied in relation to environmental cancers, with a large-scale study of over 43,000 cancer patients and over 370,000 controls reported increased risk of small cell lung cancer (OR = 2.06) and squamous cell skin cancer (OR = 1.69), indicating that individuals with this SNP are vulnerable to cancers with environmental genotoxic risk factors [49]. This study did not find an association between K3326X and upper-aero digestive tract cancers (oral cavity, oropharynx, larynx/hypopharynx, and esophagus) among Icelandic subjects. This is in contrast to an earlier study that found associated risk between K3326 and upper-aero digestive tract cancers among European, Latin American, and Indian populations [50]. The association between K3326X and cutaneous squamous cell carcinoma was confirmed by a recent large meta-analysis which embodied six international cohorts including 19,149 squamous cell carcinoma cases and 680,049 controls [51]. Evidence for the relationship of BRCA2 K3326X and environmental cancers holds up well across diverse populations. A study conducted of 190 Turkmens and 1373 controls found an increased prevalence of the variant in esophageal squamous cell carcinoma with an OR of 3.38 (95% CI = 1.97–6.91, *p* = 0.0002) [52]. In a study of Chinese esophageal cancer, the K3326X mutation was detected in just one case, suggesting that K3326X is uncommon in Henan and Hong Kong ESCC patients (wide variation exists for this mutation across populations) [53]. A genome-wide association study of 159 cases and 2707 controls including the genotyping of 1476 non-synonymous SNPs in 871 candidate genes found increased prevalence of BRCA2 K3326X in lung cancer of unspecified type (OR = 1.72, 95% CI 1.15–2.57, *p* = 0.0075) [54]. BRCA2 K3326X was assessed in a case control study of 2634 breast cancer cases from familial cancer clinics and 1996 non-cancer population controls. BRCA2 K3326X was overrepresented in cases with an OR of 1.53 (95% CI 1.00–2.34, *p* = 0.047) [55]. Additionally, a large-scale study of European populations for lung cancer risk assessed 11,348 cases and 15,861 controls from the 1000 genomes project, with a follow up confirmation of an additional 10,246 cases and 38,295 controls. The authors found BRCA2 K3326X to be significantly associated with lung cancer (OR = 2.47, *p* = 4.74 × 10^−^^20^) [56]. In this study, BRCA2 K3326X was more significantly associated with lung cancer of the squamous variety than lung adenocarcinoma (OR = 2.47, *p* = 4.74 × 10^−^^20^ and OR = 1.47, *p* = 4.66 × 10^−^^4^, respectively). The authors note that the association with squamous cancers and BRCA2 mutations is reflective of the higher mutation frequency in squamous cancers compared to adenocarcinoma. The association was not present in nonsmokers who made up a smaller portion of the cases, a limitation of the study. Finally, the study investigated the hypothesis that the K3326X variant was in linkage disequilibrium with another deleterious variant, but found no evidence of another causative mutation in those cases.

The conclusions of the above studies are briefly compiled in Table 1.

## 5. Summary of K3326X literature

**Figure 3:**
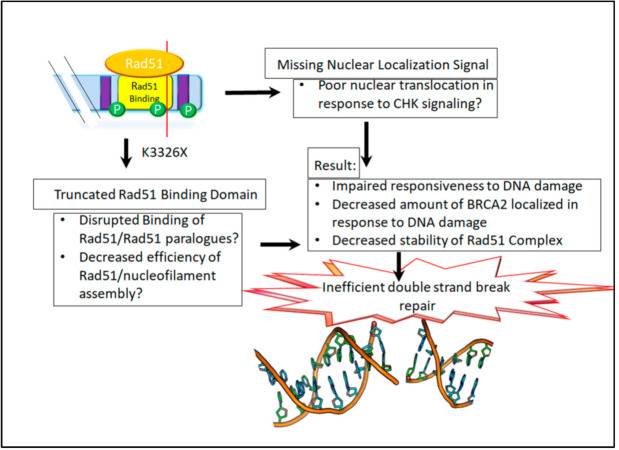
The K3326X mutation: The K3326X mutation results in the loss of the most distal 93 amino acids of the protein. This results in the loss of the final nuclear localization signal, the phosphorylated residue T3387, and the last four residues of the Rad51 binding site.

In summary, these results suggest that there exists some functional deficit by which K3326X increases individual susceptibility to cancer. A graphical representation of the hypotheses surrounding the mutation is shown in Figure 3. While heavily studied and frequently implicated in cancer development, it remains listed as “benign” in Clinvar.

Studies of the K3326X mutation are consistent in identifying moderate increased risk of environmental cancers and cancers of the hereditary breast, ovarian, and pancreatic cancer syndrome (HBOPC) family in individuals carrying the K3326X mutation. Given the nature of the C-terminal domains of BRCA2 deleted by the K3326X mutation, it is likely that this truncated form of BRCA2 is deficient in Rad51 nucleofilament assembly, nuclear localization, or both. It is also possible that the truncation could disrupt the ability of BRCA2 to protect stalled replication forks. Data in The Cancer Genome Atlas [57,58] indicate that the K3326X mutation occurred in three cases of high-grade serous ovarian cancer, each time with a second pathogenic or likely pathogenic mutation. As mentioned in the above section, it has been found with damaging mutations in ATM and Rad51D in a small cohort study [46], and with a damaging mutation in BRCA1 in an Italian cohort study [44]. Together, these studies also suggest that K3326X could increase the penetrance of another mutation or be rendered nonfunctional with additional mutational burden. Further in vitro and biochemical studies will be necessary to elucidate the exact mechanism behind this likely hypomorphic nature of this variant.

## 6. Other C-Terminal Mutations in BRCA2

Recent investigation has uncovered new evidence regarding mutations on the C-terminus of BRCA2. Many studies are limited in scope, only covering one ethnic population or mutation, and further work will be needed to generalize these results to the global patient population for better clinical utility.

da Costa e Silva, Carvalho et al. performed a molecular screening to detect causal germline variants and characterize variants of unknown significance (VUS) in a sample of the Brazilian population [59]. The screening included 21 genes related to DNA repair pathways. It involved 95 individuals who had a clinical suspicion of HBOC syndrome, and criteria for genetic risk evaluation according to the NCCN Clinical Practice Guidelines in Oncology, and a cumulative risk of BRCA1 and BRCA2 variants higher than 10%, using PennII model, in addition to a personal history of cancer. This study presented the first report of a known pathogenic variant in the Brazilian population: the p.Tyr3009Serfs*7 (c.9026_9030delATCAT) on BRCA2. A similar large scale analysis of the genes involved in ESCC in the Chinese population was conducted by Ko JM-Y et al. [53]. The study included 4517 individuals sequenced for BRCA2 germline mutations. In addition to the K3326X mutation mentioned in the previous section, another BRCA LOF variant located at the C-terminus was detected (exon27:c.10255dupT:p.I3418fs). BRCA2 variants were present at a higher frequency (3.23% vs. 0.21%) in ESCC patients in the study compared to non-TCGA East Asian ExAC controls. BRCA2 loss of function mutations were associated with high ESCC risk compared to controls (OR = 10.55, *p* = 0.00035). Most of the mutations uncovered in this study are distal to the BRC repeats and unique. The loss of function variants this study uncovered on the C-terminus were E2183X, S2219X, T3030fs, K2849fs, V3082fs, E2139X, S2984X, V3082fs, Q2065X, T3030fs, R2520X, A1689fs, R3128X, K3326X, and I3418fs. Many of these are predicted to increase the cytoplasmic BRCA2 fraction due to loss of the distal NLS. Two recurrent variants were found: G2508S (OR = 7.75, *p* = 0.026), and a second in the 3′ UTR. Most variants found in this study (68.4%) did not overlap with regions implicated in hereditary breast, ovarian, and pancreatic cancer.

Several recent small studies in specific populations have yielded information regarding risk for BRCA2 variants. A study of 55 Japanese breast cancer patients from unrelated families investigated these patients for novel SNPs in BRCA1 and BRCA2. This study uncovered 34 mutations, many of them novel, including one on the C-terminus, K2729N. The authors note that all these variants are rare and need functional investigation [60]. A study of 585 Slovak families with hereditary breast and ovarian cancer syndrome (HBOC) found evidence of pathogenic SNPs affecting the C-terminus of BRCA2 in a large-scale sequencing study. The C-terminal mutations T2197fs, T3033fs, and L3135fs were detected in one, one, and two families, respectively, with T2197fs being found only in Slovak populations [61]. In a Danish proband with a history of breast and pancreatic cancer, a deletion affecting Exon 20 was found, altering the BRCA2 protein C-terminus [62]. A study conducted in the Algerian population identified several novel missense mutations in BRCA2 as likely pathogenic, including two near the C-terminus (H2216A in the repair recombination domain, and G3086R) and several polymorphisms, including Q2384K and A2466V at the C-terminus, overrepresented in patients who tested negative for known pathogenic mutations [63]. Segregation analysis of the mutation G3076V in a Northern Italian cohort consisting of 15 families with no other known pathogenic variants suggested a pathogenicity likelihood ratio of 81,527:1, allowing the authors to classify this variant as likely pathogenic [64]. Finally, a study conducted in the Bulgarian population of breast cancer patients with a positive family history for BRCA1 and BRCA2 SNPs identified a SNP in the 3′-UTR of BRCA2 exon 27, the mononucleotide deletion in the 5′-UTR of BRCA2 exon 27, and a mononucleotide deletion in the 3′ region downstream of the gene, all three of which are unique population SNPs and could alter transcription or translation of the protein [65].

Studies like these provide the basis for the improvement of collective knowledge of genetic risk of C-terminal variants in BRCA2 and will continue to be essential to reducing the number of VUS and expanding the ability for accurate genetic counseling of hereditary cancer syndromes.

## 7. New Ways to Analyze Mutations

The search for efficient ways to classify the large number of VUS likely undergirding a significant portion of hereditary cancer risk is ongoing. New methods are necessary to enable rapid identification and interrogation of such variants in individual patients and families if necessary (reviewed in [66]). Several functional study methods have been described in detail previously (extensively reviewed in [2]). These methods include the homology-directed repair assay [67], in which functional GFP is expressed after successful HDR of an engineered allele, the centrosome amplification assay [68], which takes advantage of instability caused by BRCA2 loss, cell survival assays to DNA-damaging drugs, especially mitomycin-C [68], and the mouse embryonic stem cell (mESC) assay [69]. In this latter assay, mouse embryonic stem cells are engineered to have one defective BRCA2 allele, and a second WT conditional allele. The second allele can be switched off when a bacterial artificial chromosome containing a test allele of BRCA2 is inserted. Because BRCA2 is embryonic lethal in the null condition, this assay can quickly pinpoint fully nonfunctional VUS by complementation. Wherein functional studies are insufficient alone, novel computational algorithms can further interrogate VUS. Below is a selection of new functional tests and other methods that have recently been applied to BRCA2. As each method discussed below is experimental, and for conciseness, we do not report the totality of the results. Further validation of these methods will be needed before they can be used to provide clinical information about variants.

A large portion of inherited cancer risk caused by BRCA2 mutations may be accounted for by noncoding mutations [70]. Furthermore, many moderate impact missense and noncoding mutations in BRCA2 may be most useful integrated into genomic risk prediction (reviewed in [71]) for greater clinical utility in patient counseling. Not all pathogenic variants are equal in their cancer predisposition potential either. Differences in relative risk for known pathogenic SNPs in BRCA2 have been previously demonstrated, and using this knowledge, patients can be enabled to make more informed decisions about their future health, with a UK study showing that the overall breast cancer SNP risk and age of onset can vary significantly between pathogenic BRCA2 SNPs [72]. The predictive ability of SNPs is hypothesized to be higher if demographic and environmental data is included [73]. Alternatively, there is evidence that some information regarding SNPs may be simply semantic. BRCA mutation carriers are known to have a higher risk of contralateral breast cancer, about 3% over 20 years after the initial cancer. However, it was found that SNP testing did not add any additional information about patient risk that may be useful in recommending contralateral mastectomy [74].

Considering the rarity of most missense variants in both general populations and cancer patients, and the limited statistical significance in case-control studies to classify these variants as pathogenic or benign, Ikegami et al. conducted a high throughput method using a BRCA2-null cell line (DRD1 BRCA2−/−) into which a BRCA2 vector with a barcoded variant and transposase are transfected. After drug treatment, the proportion of each variant transfected in the surviving cells is used to interpolate information about the pathogenicity of the individual variants. The authors have named this method the Mano-B method and employed 3 PARP inhibitors and cisplatin in their drug screen. This model was then further refined by Bayesian inference for higher accuracy. The authors attempted to classify the pathogenicity of 186 BRCA2 VUS. Their results suggested that the 126 VUS in the study should be classified as 54 “neutral/likely neutral”, 23 as “intermediate”, and 37 as “deleterious/likely deleterious” [75].

A different approach to high-throughput testing of VUS in BRCA2 was conducted by Mesman et al. using the mESC based assay mentioned above. In this study, a mouse embryonic stem cell line was used with BRCA2 variants added via bacterial artificial chromosomes. The resulting cell lines were then assessed for variant functionality via cell cycle analysis, homology dependent repair assay, complementation, and drug sensitivity. Using a set of known pathogenic/nonpathogenic variants as a validation set, the authors assessed a total of 43 VUS, 36 of them in the C-terminus as defined in this review. Among the variants, the three assessment parameters varied, with many able to perform complementation, but showing deficiency in HDR, drug sensitivity, or both. The results thus show that many of the tested variants may have an intermediate or mechanism-dependent phenotype, thus requiring further study [76]. Likewise, this methodology was also applied by Biswas et al. to characterize a panel of 88 VUS in BRCA2, with complementation in the mESC cell line used as a measure of impact in addition to drug sensitivity with a panel of six DNA-damaging agents. The authors combined these data with a novel VarCall model to classify each variant with a probability of normal function [77].

Computational methods show promise for rapidly generating and testing hypotheses about missense mutations. As more is learned about the protein and as algorithms are refined, these methods will improve and may eventually replace time consuming and technically difficult wet lab techniques. However, as of this writing, these are not sensitive or specific enough to operate without wet lab or population-based study confirmation [78]. The computational algorithm MH-BRCA with MH Guide has been employed to aid in clinical interpretation of data from likely hereditary breast and ovarian cancer families in Japan. This toolset showed high concordance with known variants in validation and could be used for the assessment of novel variants. However, disagreement with the tool’s ACMG classification and the ENIGMA database are a source of potential problems. The authors further use this tool to identify likely pathogenic variants and predict the success of PARP inhibitor therapy in a retroactive study [79].

Specific mutations can be investigated in individual small scale studies via a combination of computational and wet lab methods. Such was the case for R2787H in BRCA2, which was classified by a combination of computational algorithms and wet lab docking studies, determining that this mutation was most likely pathogenic. Under study, the mutation showed reduced stability in binding with ssDNA compared to WT BRCA2 in both computational and biochemical experiments [80]. Similarly, in response to a case of fibrocystic dysplasia in young women, leading to the identification of P2767S as a variant of interest, Esposito et al. performed in silico and in vitro functional analysis on this variant. The VUS occurs in the DNA binding domain of the BRCA2 C-terminus, and was found to be heavily conserved between species, and predicted to be critical to proper folding and stability of the domain. This hypothesis was tested by in vitro ssDNA binding assays, and it was shown that P2767S disrupted the ability of BRCA2 to bind to damaged ssDNA [81].

Finally, it has been proposed that “coldspots” exist within proteins, defined as regions in which no pathogenic missense variants are present because of the inessential nature of said region. While it is likely that location in a coldspot is not definitive evidence of benign nature, incorporating coldspots into prediction algorithms could improve their accuracy and clinical utility [82]. Hotspots may also exist and could be uncovered in much the same way. A veterinary investigation of mammary tumors in dogs found evidence that SNPs in exons 24 and 27 of BRCA2 may be associated with cancer development [83]. Thus, classification of benign and pathogenic SNPs across the protein could be used to show regions of the protein of clinical concern.

## 8. Conclusions

The C-terminal region of BRCA2 contains the most conserved amino acids between metazoans and fungal homologs, although there is only one stretch within last 93 amino acids in common between human and chicken BRCA2 [84]. This region (last 93 amino acids) contains a critical phosphorylation site and is associated with RAD51 binding. Therefore, if we are to evaluate the pathogenicity of a single amino acid change, it cannot be determined suitable for clinical application using algorithms employing conservation as the major factor. High-throughput functional tests will need to be developed to support precision medicine so as to fully understand the genetic risk of cancer or potential therapeutic strategies. In addition, we should rely on multiple functional tests appropriate for each gene product to have sufficient information to provide comprehensive analysis. Other than SNPs in coding regions of cancer risk genes, additional genetic regions such as 5′ and 3′ untranslated regions and sequences critical for proper gene expression must be addressed.

## Figures and Tables

**Table 1:** K3326X cancer associations: The studies of BRCA2-K3326X presented in this review are summarized here, in reference order, and with cancer type, *p*-value, study population size, and recommendations from the authors of the study. All studies found significant odds ratios above 1 but less than 5. The authors varied in their assessment of the variant but most recommended further investigation.

Reference	Cancer Type, [Odds Ratio]	*p*-Value	Population (Number)	Recommendation
[42,43]	Breast (1.28)	5.9 × 10^−6^, 3.8 × 10^−3^, 3.4 × 10^−5^, 4.1 × 10^−5^	70,000 cases	Expanded counseling
	Invasive ovarian (1.26)		80,000 controls	
	Serous ovarian (1.46)		-	
	ER-negative breast (1.5)		-	
[44]	Early onset	N/A	Small (case study)	Further evaluation
[46]	Ovarian cancer (4.95)	<0.01	48	Treat as low risk pathogenic
[47]	Familial pancreatic (4.84)	<0.01	114 familial pancreatic cancer	None
			115 environmental control	
			125 controls	
[48]	Pancreatic ductal adenocarcinoma (1.78)	0.0012	2935 cases	None
			5626 controls	
[49]	Small cell lung (2.06)	9 × 10^−4^, 4.2 × 10^−4^, 1.2 × 10^−4^, 1.6 × 10^−5^	43,641 cases 370,971 controls	Vulnerability to environmental cancers
	Squamous skin (1.69)			
	Lung cancer (1.54)			
	All cancers (1.23)			
[50]	Upper aero digestive tract (2.53)	3 × 10^−10^	5942 cases	Warrants further investigation
			8086 controls	
[51]	Cutaneous Squamous cell carcinoma (2.29)	1.0 × 10^−6^	Meta-analysis of 19,149 cases, 680,049 controls	Variant likely implicated in skin cancer development
[52]	Esophageal squamous cell carcinoma (3.38)	0.0002	190 cases	None
			1373 controls	
[53]	Esophageal cancer	N/A	2276 cases	Rare variant in Henan and Hong Kong ESCC patients
			2058 controls	
[54]	Lung Cancer (1.72)	0.0075	1529 cases	Low penetrance alleles contribute to risk
			2707 controls	
[55]	Breast Cancer (1.53)	0.047	2634 cases	Variant is not neutral, should be included in SNP panels for evaluating risk
			1996 controls	
[56]	Squamous lung cancer (2.47)	4.7 × 10^−20^	10,246 cases	K3326X may have a direct effect on lung cancer development
			38,295 controls	

## Data Availability

No new data were created or analyzed in this study. Data sharing is not applicable to this article.
